# 13715 Leveraging Clinical Research Infrastructure to Correct Identification of Patients’ Primary Care Physicians in a Community Hospital

**DOI:** 10.1017/cts.2021.722

**Published:** 2021-03-30

**Authors:** Laura Magda, Emily Perish, Darielle Sherrod, Lisa Dubin, Shavon Clark-Howard, Ashley Aguilar, Mary Cormier, Joshua Smith, David Meltzer

**Affiliations:** University of Chicago Medicine

## Abstract

ABSTRACT IMPACT: Correctly identifying and documenting patients’ primary care physicians in community hospital settings may improve clinical care and coordination for patients, potentially leading to a reduction in hospitalizations, while simultaneously increasing patient education and expanding the limits of electronic health record systems. OBJECTIVES/GOALS: Clinical research infrastructure can be leveraged to detect inaccuracies in primary care physician (PCP) identification and documentation in a community hospital. As a result, collaboration between clinical research and hospital clinical operations can produce long-term solutions required by patient-centered, learning health care systems. METHODS/STUDY POPULATION: Hospitalized patients at a community hospital were asked to verify the name of their PCP. The PCP name given by the patient was then compared to the PCP on file in the EHR system. A corrected list of PCP names for each patient was sent by a clinical research program to hospital management on a weekly basis and used to update the EHR. RESULTS/ANTICIPATED RESULTS: A total of 272 hospitalized patients were screened on the basis of eligibility and asked to verify their PCP name. Overall, 35.3% (N=96) of patients had incorrectly listed PCPs in the EHR system. DISCUSSION/SIGNIFICANCE OF FINDINGS: Accurate PCP identification processes may enable broader clinical communication and potentially reduce future hospitalizations by improving coordination of care. The benefits of collaboration between research and clinical activities may provide an opportunity to justify greater investment in clinical research in community settings.

